# Pharmacists’ Perspectives on Interprofessional Collaboration with Physicians in Poland: A Quantitative Study

**DOI:** 10.3390/ijerph18189686

**Published:** 2021-09-14

**Authors:** Łucja Zielińska-Tomczak, Magdalena Cerbin-Koczorowska, Piotr Przymuszała, Natalia Gałązka, Ryszard Marciniak

**Affiliations:** 1 Department of Medical Education, Poznan University of Medical Sciences, 7 Rokietnicka St., 60-806 Poznan, Poland; mcerbin@ump.edu.pl (M.C.-K.); pprzymuszala@ump.edu.pl (P.P.); rmarcin@ump.edu.pl (R.M.); 2 Students’ Scientific Club of Medical Education, Department of Medical Education, Poznan University of Medical Sciences, 60-806 Poznan, Poland; s83002@student.ump.edu.pl

**Keywords:** interprofessional collaboration, physician–pharmacist collaboration, collaboration barriers, community pharmacists

## Abstract

Over the years, many studies have emphasized the pharmacist’s importance as part of the patient care team. Still, the interprofessional collaboration between physicians and pharmacists in their everyday work seems rare. Therefore, this study aimed to investigate the types of contact between them, possible mutual collaboration, and barriers to implementation. This study was conducted from April to August 2020. The study group included licensed pharmacists working in community pharmacies in Poland (*n* = 207). The results show that, according to the respondents, physician–pharmacist contact mainly concerns formal aspects, such as correcting prescription errors. They occasionally communicate for other matters, such as consultation regarding drug availability and drug dosage. However, when asked to divide responsibilities between them and physicians, pharmacists indicate areas that should involve interprofessional collaboration, e.g., monitoring adverse drug reactions, analysis of multi-drug therapy, and checking the regularity of taking medications. They indicated the lack of specific collaboration rules, limited willingness to establish relationships and low mutual respect and trust among existing barriers. It is worth considering the possibility of overcoming these barriers provided by interprofessional education in order to develop communication skills and build relationships based on respect.

## 1. Introduction

Interprofessional collaboration in health care has been in place for years, and interprofessional teams are common in many parts of the world [1,2,3,4]. However, in some health care systems, the accomplishment of real interprofessional collaboration, in which all health care team members are engaged as equals and contribute their different knowledge and experience to achieve common clinical goals might seem an elusive idea. The physician’s dominant role as a clinical leader seems very firmly entrenched in the hierarchical health care system. On the other hand, some health care professionals are not even viewed as potential team members [5]. However, interprofessional care may become a factor in reducing the existing disproportion between the public demand for medical services and the health care system’s limited human resources [6]. Increasing problems include growing numbers of patient health concerns with a simultaneous increase in their complexity [7], as well as an overall longer average life expectancy, resulting in a significantly higher risk of chronic diseases [8].

Interprofessional care enables comprehensive care. Interprofessional care can be described as different health care professionals working together toward a common purpose—improving patient health [9]. Studies dedicated to the collaboration between physicians and pharmacists show that it can contribute to faster recovery and improvements in results (e.g., glucose control and blood pressure) [10,11,12,13,14,15,16,17]. Pharmacist involvement can also improve the quality of patient care by facilitating the identification of drug interactions and preventing the use of unsafe or ineffective treatment regimens [18,19]. Moreover, although the traditional model with a doctor diagnosing diseases and prescribing drugs that a pharmacist prepares and distributes seems insufficient in the modern health care system, the representatives of both professions still do not cooperate effectively in some parts of the world [20,21]. Bradley et al. [22] proposed a three-level model to assess the collaboration between physicians and pharmacists: level one—isolation, level two—communication, and level three—cooperation. In Poland, for example, the relationship between pharmacists and physicians is described to be on the level of isolation. This is evidenced by geographical separation and the dominant role played by the doctor. Pharmacists in Poland do not provide pharmaceutical care, and their role is often limited to formal activities [23].

Despite the growing awareness of benefits arising from pharmacist involvement [10,11,12,13,14], the establishment of interprofessional relationships is still being challenged by conflicts and communication problems [24]. Therefore, it seems necessary to examine the current state of physician–pharmacist collaboration and the types of contact between them to identify possible solutions for the development of interprofessional care. Consequently, this study aimed to assess pharmacists’ opinions, experiences, and expectations toward interprofessional collaboration.

## 2. Materials and Methods

This study was conducted from April to August 2020. The study group included licensed pharmacists working in community pharmacies in Poland. Participants were recruited from a diversified sample of pharmacies in terms of their geographical distribution (different parts of Poland) and urbanization levels (towns and villages).

In the interest of high content validity, the questionnaire used in this study was developed based on a previously conducted qualitative study [25]. Following the results in the study, appropriate thematic blocks were distinguished, which were later used to form questions and answer options. The obtained questionnaire consisted of 10 questions regarding the collaboration between physicians and pharmacists (for instance, pharmacists’ opinions, experiences, and expectations toward interprofessional collaboration) and 6 questions regarding the characteristics of the respondent (profession, work experience, places of work, foreign internship, and specialization). The questionnaire was preceded by instructions for respondents about the aim of this study and how to fill out the questionnaire.

Before sending it to participants, the questionnaire was pretested on a sample of three pharmacists in terms of comprehensibility and absence of suggestive questions. The suggestions made by the pharmacists during the pretesting procedure resulted in minor wording changes in some questions to increase their understandability. The responses of the pharmacists participating in the pretest were not included in further analysis.

The online version of the questionnaire was created using the researchonline.pl portal [26] and sent to a total of 3600 community pharmacies by e-mail. The mailing database was created based on data obtained from the Polish register of pharmacies [27].

The data obtained were analyzed with the Statistica PL 13.3 software (StatSoft) using the chi-squared test (χ^2^), Fisher’s exact test, the Mann–Whitney U test, and the Kruskal–Wallis test with post hoc Dunn’s test. Statistical significance was assumed at *p* < 0.05.

## 3. Results

### 3.1. Study Group

The questionnaire was opened 680 times and completed by 207 pharmacists working in community pharmacies. Participants worked in the profession for an average of 14.0 ± 10.3 years, and nearly 60% had less than 15 years of work experience. The study group was comprised of pharmacists employed in large, medium, and small towns and to a smaller extent in rural areas, which is consistent with the distribution of pharmacies in Poland [27]. The distinction between large, medium, and small towns was made according to the definitions adapted by the Polish Central Statistical Office (small towns—below 20,000 inhabitants; medium towns—20,000–100,000 inhabitants; large towns—100,000 and more inhabitants) [28]. Among the studied group, 30% of the respondents (*n* = 62) had a specialization. In Poland, it is possible for pharmacists to specialize in particular areas (e.g., community pharmacy, hospital pharmacy, and clinical pharmacy). However, having a specialization is not required to work in the profession [29]. The characteristics of the studied population are presented in Table 1.

### 3.2. Pharmacists’ Opinions on Interprofessional Collaboration

The vast majority of pharmacists considered collaboration with physicians a necessity (n_4+5_ = 204; 99.0%), and only slightly less assessed it as feasible (n_4+5_ = 173; 83.7%). Still, according to 48.3% of the respondents (n_1+2_ = 100), the current level of cooperation with doctors is bad or very bad. Only three respondents described the collaboration as excellent. The current state of partnership with physicians was better assessed by pharmacists with specialization (*p* = 0.031) (Table 2). Respondents mostly positively assessed their attitude toward cooperation. However, there was a significant difference in the intensity of that opinion, dependent on an individual’s participation in internships abroad (*p* = 0.011). Unfortunately, given the small number of respondents who gained professional experience abroad, caution is advised in the interpretation of this observation.

The Kruskal–Wallis test showed that perception of possible collaboration differs depending on the workplace’s degree of urbanization. Dunn’s post hoc test verified that groups with statistical differences are pharmacists in rural areas and cities with more than 100,000 inhabitants.

### 3.3. Pharmacists’ Experiences with Interprofessional Collaboration

As presented in Table 3, in the respondents’ experience, most common physician–pharmacist occurs in the correction of medical prescription errors. Other reasons for contact constitute consultations regarding drug availability at the pharmacy and correct drug dosage, but they were indicated noticeably less frequently. Other occasions for contact, e.g., consultations on choosing a drug substance, side effects, drug interactions, and medical devices, are very rare. Pharmacists with foreign experience tend to contact doctors more often on matters related to compounding medication than pharmacists who have not gained professional experience abroad (*p* = 0.018). Again, we recommend caution during interpretation due to the small sample of respondents with a foreign internship.

**Figure 1:**
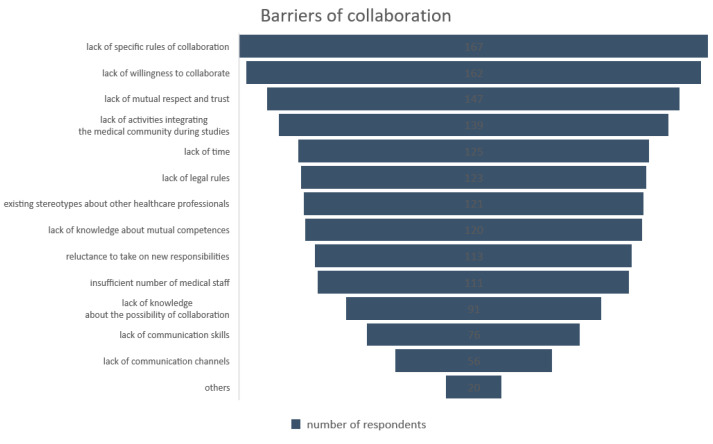
Barriers of collaboration.

Among the barriers, more than half of the surveyed pharmacists indicated the lack of specific collaboration rules, limited willingness to establish relationships, and low mutual respect and trust. Reasons cited for the difficult contact between physicians and pharmacists also included a lack of activities integrating the medical community during studies, a lack of time, and insufficient legal regulations (Figure 1). Some pharmacists gave additional reasons impeding the establishment of collaboration, which were not anticipated in the questionnaire, such as inadequate knowledge about mutual competencies or fear of losing competencies.

*R111: Belief that cooperation could interfere with the competencies of one of the parties.*

*R7: Lack of knowledge (awareness) of doctors about the knowledge of pharmacists.*

*R26: arrogance and underestimating the second profession and the competence of health care workers.*

In the opinion of some pharmacists, certain physicians seem to perceive themselves as superior, which makes cooperation difficult. Moreover, they believe that some doctors may not even see pharmacists as partners, marginalizing their role to sellers. Additionally, respondents also report low self-esteem.

*R51: Sense of superiority of physician’s profession over the rest of healthcare professions.*

*R140: Most physicians treat pharmacists as salespeople.*

*R142: Physicians do not treat pharmacists as partners but rather as their subordinates because they think that only they [physicians] can cure, and pharmacists do not treat anyone.*

### 3.4. Pharmacists Expectations toward Interprofessional Collaboration

**Figure 2:**
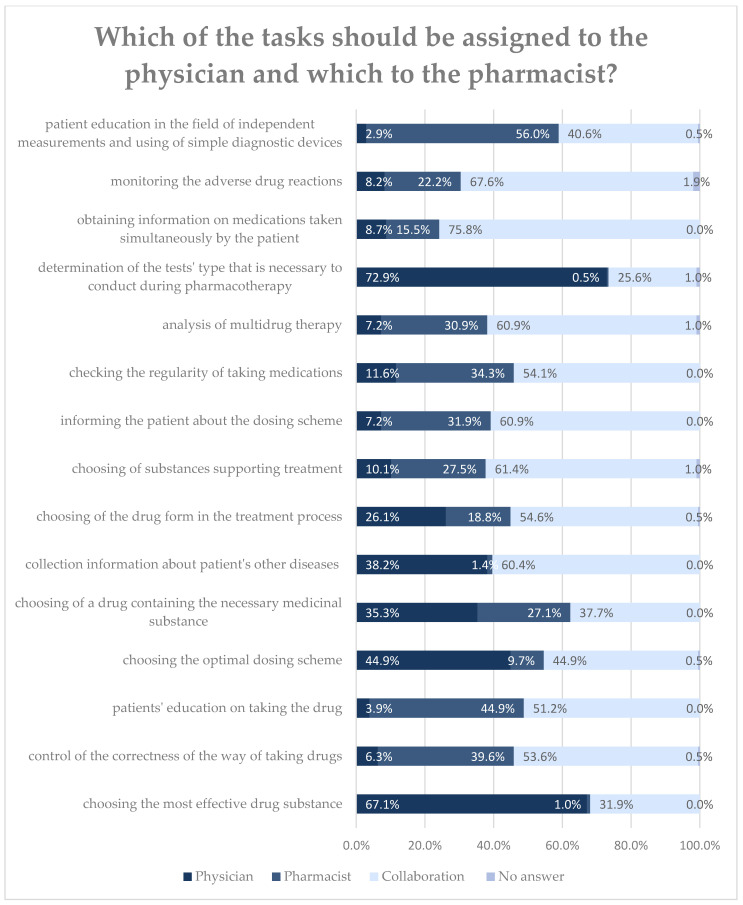
The proposed division of tasks between the physician and the pharmacist.

Pharmacists indicate that themselves as responsible for educating patients on using simple diagnostic devices (*n* = 116; 80.2%). On the other hand, they indicate that physicians are responsible for determining the type of tests necessary in the course of pharmacotherapy (*n* = 151; 73.0%) and choosing the most effective drug substance for a given disease (*n* = 139; 67.2%). However, in most tasks, they answered that interprofessional collaboration is necessary. Pharmacists with specialization perceive the task of choosing a preparation containing the treatment substance differently (*p* = 0.008). In their opinion, the physician should be responsible for selecting the preparation (*n* = 33; 53.2%), while pharmacists without specialization see the possibility of cooperation in this respect (*n* = 59; 40.9%). Differences are also observed in the approach of pharmacists with professional experience gained abroad in analysis of multi-drug therapy. They believe that this is in the pharmacist’s competence (*n* = 10; 62.5%). The remaining pharmacists want cooperation with doctors in this regard (*n* = 122; 63.9%). However, due to the small sample of respondents with a foreign internship, this observation should be interpreted with caution. Detailed information on the areas is presented in Figure 2.

**Figure 3:**
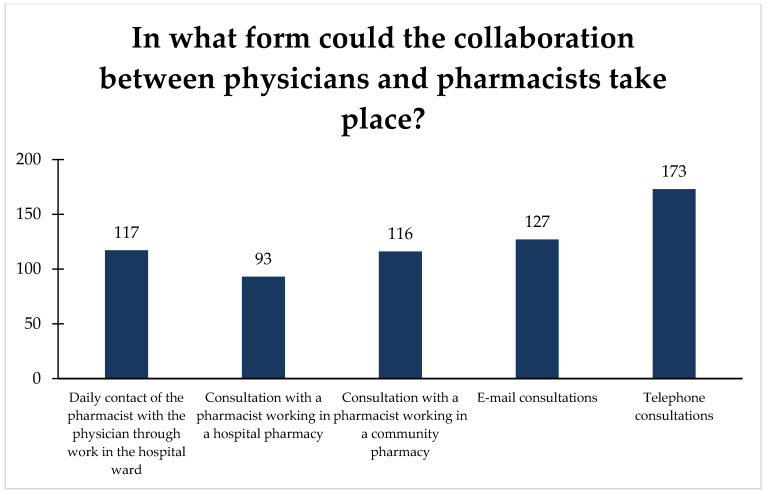
Proposed forms of collaboration. Answer the question: In what form could the collaboration between physicians and pharmacists take place?

Pharmacists were also able to indicate how collaboration with physicians could take place. Most pharmacists have chosen to contact physicians by phone or e-mail, followed by daily contact through joint work in the ward and consultations in pharmacies (Figure 3).

## 4. Discussion

The studies carried out so far show that the collaboration between representatives of various medical professions, both in hospital and ambulatory care, results in increased patient satisfaction and acceptance of the therapy [14]. They also provide evidence for the importance of interprofessional activities in improving the effectiveness and safety of the treatment, including the management of chronic diseases [30]. Additionally, various studies in Poland emphasize that both pharmacists and students of pharmacy expect to expand their professional role and see themselves as competent specialists in the field of pharmacotherapy [31,32,33].

However, as our results show, this evidence still does not translate into practice in Poland. This study shows that contact between Polish physicians and pharmacists is still occasional and limited mainly to correcting prescription errors. As indicated by Alkhateeb et al. [34], a relationship based solely on formal issues is not conducive to building an interprofessional relationship and reduces doctors’ interest in establishing future cooperation. Lack of contact in pharmacotherapy matters may be caused by the lack of knowledge about mutual competencies and the fear of losing own competencies resulting from misunderstandings of mutual roles [33,35,36]. Additionally, the fear of losing ‘professional territory’ makes it difficult to build partnership relations [37].

Although pharmacists declare their positive attitude to cooperation with doctors, they list the lack of willingness to cooperate as one of the barriers to establishing interprofessional relationships. Żak [21] showed in his study that pharmacists emphasize that the lack of effective cooperation with doctors is a significant barrier to pharmaceutical care. He also points out that the negative attitude of doctors to cooperation may limit this service. Similarly, a study by van Mill et al. [38] showed that pharmacists’ and other health care professionals’ attitudes constitute one of the two most important barriers to the introduction of pharmaceutical care in European countries. Meanwhile, Ajzen’s theory of planned behavior indicates that attitude is one of the three determinants influencing intention and, as a result, performing given behavior [39].

Among the identified factors influencing the development of the correct collaboration are mutual respect, trust, communication, and knowledge of each other [40]. Similarly, in this study, pharmacists identified the lack of these characteristics among barriers to implementing the collaboration. Noteworthy, 71% of respondents mentioned the lack of mutual trust and respect, and almost 58% indicated the lack of knowledge about mutual competencies. On the other hand, fewer respondents indicated a lack of communication skills (36.7%) or a lack of available communication channels (27%). Still, it should be mentioned that Żak [21] pointed to the absence of an integrated IT system on the physician–pharmacist line as a cause of the lack of more effective cooperation opportunities.

Simultaneously, there are other barriers to cooperation, such as the lack of time to develop collaboration. Both Goldstone et al. [41] and Żak [21] indicate that this is caused by staff shortages and the number of duties reported by both doctors and pharmacists.

What is more, there is a lack of described standards or guidelines of cooperation between both professions in Poland, which may, in turn, translate into a negative approach to collaboration between physicians and pharmacists [21]. However, recently in Poland, actions were undertaken to change this situation, such as the demand of the Supreme Medical Chamber to clarify the aspects of cooperation [42] or the adoption of the Act on the Pharmacist’s Profession [43], which legally regulates the competences of pharmacists and provides grounds for initiating their cooperation with doctors.

In this study, pharmacists indicate medical areas in which collaboration with a physician would be possible, including monitoring side effects of drugs, analysis of multi-drug therapy, patient education, or checking the regularity and correct way of taking medications. Many of these were also previously described in studies from both Poland and other countries. For instance, previously conducted research shows that Polish pharmacists are prepared and open to getting involved in patient health education [44,45]. A similar increase in the involvement of pharmacists in patient education is also observed in other countries [46]. Further, it is worth emphasizing that joint International Pharmaceutical Federation and World Health Organization guidelines on good pharmacy practice [47] also place the pharmacist as the medical partner responsible for identifying alarming symptoms and educating patients. Furthermore, other studies also show that pharmacists are ready to expand the scope of their practice and perform roles in addition to engaging in the design and supervision of pharmacotherapy, such as identifying and preventing prescription errors or suggesting non-prescription medications to patients [48,49,50,51]. Noteworthy, patient education is perceived as an area where cooperation between representatives of both professions is particularly desirable [52].

Nevertheless, it seems that pharmacists still rarely contact doctors, thus maintaining the traditional model of physician–pharmacist contact [20]. The literature distinguishes three stages of interprofessional relationships: isolation, communication, and cooperation [40]. Unfortunately, reports from different countries still present the model as corresponding to the first stage, with separation of work and isolation of pharmacists and physicians [34,48,49,53,54,55]. Meanwhile, pharmacists pay attention to the possibility of contacting a physician, emphasizing the use of information and communication technology. The results of previous studies also show that it should be developed and promoted in collaboration between the doctor and pharmacist [56,57,58]. 

Last but not least, although it did not appear among the results of this study, interprofessional education (IPE) can provide opportunities to develop cooperation between doctors and pharmacists. IPE is an educational model involving training in teams in which two or more professionals learn with each other, from each other and about each other [59]. Participation in IPE enables learners to deepen their knowledge, develop mutual respect, communicate effectively and learn about each other’s competencies [60]. IPE teaches understanding of individual specialists’ roles and develops a willingness to cooperate, one of the basic building blocks of a proper interprofessional relationship. Activities of this nature affect the behavior change in the interprofessional group. They teach how to solve problems together, improve the use of communication techniques such as active listening [61,62], and broaden knowledge and learn about colleagues’ competencies [40]. As previous research suggests, including IPE in postgraduate training may prove beneficial [63]. While many interventions have been designed to enhance interprofessional collaboration, a systematic review by Bollen et al. [64] concluded that most of the research has focused on the educational effectiveness of these initiatives, and too little attention has been paid to behavioral changes. In light of this research, it is planned to check how IPE classes affect attitudes and establish cooperation.

### Limitations

We acknowledge that this study has limitations. First of all, the study group consisted of pharmacists working in community pharmacies, and it may be worth extending this study to learn about perspectives of pharmacists working in other places, e.g., hospital pharmacies. Another limitation is the low response rate. Although we sent invitations to participate to 3600 pharmacies, only 207 pharmacists completed the survey. Further, we cannot exclude the impact of the COVID-19 pandemic on the collected results. During this study, pharmacists were burdened with numerous professional duties, potentially affecting their involvement in the research and opinions on the topic under study. Finally, we cannot rule out bias in the first and second researchers, who, as licensed pharmacists, have their own views on the subject. Therefore, researchers with different professional backgrounds (PP—physician, NG—pharmacy student, and RM—physician) were included in the study group of researchers.

## 5. Conclusions

Pharmacists and doctors in Poland contact each other, mainly on formal matters, such as correcting prescription errors. This contact may contribute to the deepening of one of the barriers—the lack of willingness to collaborate. Other barriers, such as the lack of established standards of cooperation, respect, self-confidence, and knowledge of mutual competencies, also hinder the development of interprofessional relations. The possibility of overcoming these barriers with IPE is worth considering, as it develops communication skills and teaches how to build proper relationships based on respect and trust.

## Figures and Tables

**Table 1:** The characteristics of respondents.

	*n*	Percent, %
Number of respondents	207	100.0
Workplace ^1^		
village	19	9.2
small town	55	26.6
medium town	65	31.4
large town	68	32.9
Seniority (years)		
≤5	62	30.0
6–15	60	29.0
16–25	52	25.1
≥26	33	15.9
Foreign internship		
Yes	16	7.7
No	191	92.3
Specialization		
Yes	62	30.0
No	145	70.0

Workplace ^1^: defined based on definitions from the Polish Central Statistical Office (small towns—population below 20,000 inhabitants; medium towns—population 20,000–100,000 inhabitants; large towns—population above 100,000 inhabitants) [28].

**Table 2:** Pharmacists’ opinions on interprofessional collaboration.

Variables		Results								
		Σn	n_1+2_	n_3_	n_4+5_	Dominant	Median	Lower Quartile	Upper Quartile	*p*-Value
How is the collaboration between pharmacists and physicians currently shaped? (response options on a scale from 1—cooperation is very bad to 5—cooperation is very good)										
		182	100 (55.0%)	43 (23.6%)	39 (21.4%)	2	2	2	3	
Specialization	No	127	77 (60.6%)	27 (21.3%)	23 (18.1%)	2	2	2	3	*p* = 0.031
	Yes	55	23 (41.8%)	16 (29.1%)	16 (29.1%)	2–3	3	2	4	
Workplace ^1^	village	17	10 (58.8%)	3 (17.7%)	4 (23.5%)	2	2	2	3	ns
	small town	49	21 (42.9%)	15 (30.6%)	13 (26.5%)	2	3	2	4	
	medium town	53	29 (54.7%)	14 (26.4%)	10 (18.9%)	2	2	2	3	
	large town	63	40 (63.5%)	11 (17.5%)	12 (19.1%)	2	2	2	3	
Foreign internship	No	169	89 (52.7%)	42 (24.9%)	38 (22.5%)	2	2	2	3	ns
	Yes	13	11 (84.6%)	1 (7.7%)	1 (7.7%)	2	2	2	2	
Is collaboration between a pharmacist and a physician possible? (response options on a scale from 1—definitely not to 5—definitely yes)										
		207	19 (9.2%)	15 (7.2%)	173 (83.6%)	4	5	4	5	
Specialization	No	145	13 (9.0%)	11 (7.6%)	121 (83.4%)	4–5	4	4	5	ns
	Yes	62	6 (9.7%)	4 (6.4%)	52 (83.9%)	5	5	4	5	
Workplace ^1^	village	19	4 (21.1%)	3 (15.8%)	12 (63.1%)	4	4	3	5	*p* = 0.026
	small town	55	5 (9.1%)	2 (3.6%)	48 (87.3%)	4	4	4	5	
	medium town	65	5 (7.7%)	6 (9.2%)	54 (83.1%)	5	4	4	5	
	large town	68	5 (7.4%)	4 (5.8%)	59 (86.8%)	5	5	4	5	
Foreign internship	No	191	18 (9.4%)	14 (7.3%)	159 (83.3%)	5	4	4	5	ns
	Yes	16	1 (6.3%)	1 (6.3%)	14 (87.5%)	5	4	4	5	
Assess your attitude to collaboration between a physician and a pharmacist (response options on a scale from 1—very reluctant to 5—very willing)										
		198	24 (12.1%)	18 (9.1%)	156 (78.8%)	5	5	4	5	
Specialization	No	140	19 (13.6%)	15 (10.7%)	106 (75.7%)	5	5	4	5	ns
	Yes	39	5 (12.8%)	4 (10.7%)	30 (76.9%)	5	5	4	5	
Workplace ^1^	Village	16	2 (12.5%)	7 (43.8%)	7 (43.8%)	4–5	4	4	5	ns
	small town	45	9 (20.0%)	8 (17.8%)	28 (62.2%)	5	4	3	5	
	medium town	61	10 (16.4%)	6 (9.8%)	45 (73.8%)	5	5	4	5	
	large town	67	3 (4.5%)	5 (7.5%)	59 (88.0%)	5	5	4	5	
Foreign internship	No	184	24 (13.0%)	19 (10.7%)	141 (76.6%)	5	4	4	5	*p* = 0.011
	Yes	15	0 (0.0%)	0 (0.0%)	15 (100.0%)	5	5	5	5	

Σn—total number of respondents. n_1+2_—number of respondents who selected options 1 or 2 on the scale in response to a given question. n_3_—number of respondents who selected option 3 on the scale in response to a given question. n_4+5_—number of respondents who selected option 4 or 5 on the scale in response to a given question. ns—no statistically significant differences. *Workplace* ^1^: were prepared on the basis of definitions from the Polish Central Statistical Office (small towns—population below 20,000 inhabitants; medium towns—population 20,000–100,000 inhabitants; large towns—population above 100,000 inhabitants) [28].

**Table 3:** The purpose of contact with physicians.

Variables	Results							
	How Often in the Last Year Have You Made Contact with Physicians Regarding (1—Very Rarely, 5—Very Often)							
	Σn	n_1+2_	n_3_	n_4+5_	Dominant	Median	Lower Quartile	Upper Quartile
formal aspects								
correction of a prescription error	207	17 (8.2%)	36 (17.4%)	154 (74.4%)	5	4	3	5
consultation on drug availability at the pharmacy	207	94 (45.4%)	53 (25.6%)	60 (29.0%)	1	3	1	4
substantive aspects								
consultation on drug dosage	207	150 (72.5%)	40 (19.3%)	17 (8.2%)	1	2	1	3
consultation on the choice of a drug substance	207	189 (91.3%)	14 (6.8%)	4 (1.9%)	1	1	1	1
consultation on drug interactions	197	188 (95.5%)	5 (2.5%)	4 (2.0%)	1	1	1	1
consultation on side effects	207	202 (97.6%)	2 (2.0%)	3 (1.4%)	1	1	1	1
prescription drug consultation	182	127 (69.8%)	47 (25.8%)	8 (4.4%)	1	2	1	3

Σn—total number of respondents. n_1+2_—number of respondents who selected options 1 or 2 on the scale in response to a given question. n_3_—number of respondents who selected option 3 on the scale in response to a given question. n_4+5_—number of respondents who selected option 4 or 5 on the scale in response to a given question.

## Data Availability

The data used to support the findings in this study are available from the corresponding author upon request.
